# Exploring the Frontiers of Neuroimaging: A Review of Recent Advances in Understanding Brain Functioning and Disorders

**DOI:** 10.3390/life13071472

**Published:** 2023-06-29

**Authors:** Chiahui Yen, Chia-Li Lin, Ming-Chang Chiang

**Affiliations:** 1 Department of International Business, Ming Chuan University, Taipei 111, Taiwan; 2 Department of Life Science, College of Science and Engineering, Fu Jen Catholic University, New Taipei City 242, Taiwan

**Keywords:** EEG, fMRI, neurological disorders, tDCS

## Abstract

Neuroimaging has revolutionized our understanding of brain function and has become an essential tool for researchers studying neurological disorders. Functional magnetic resonance imaging (fMRI) and electroencephalography (EEG) are two widely used neuroimaging techniques to review changes in brain activity. fMRI is a noninvasive technique that uses magnetic fields and radio waves to produce detailed brain images. An EEG is a noninvasive technique that records the brain’s electrical activity through electrodes placed on the scalp. This review overviews recent developments in noninvasive functional neuroimaging methods, including fMRI and EEG. Recent advances in fMRI technology, its application to studying brain function, and the impact of neuroimaging techniques on neuroscience research are discussed. Advances in EEG technology and its applications to analyzing brain function and neural oscillations are also highlighted. In addition, advanced courses in neuroimaging, such as diffusion tensor imaging (DTI) and transcranial electrical stimulation (TES), are described, along with their role in studying brain connectivity, white matter tracts, and potential treatments for schizophrenia and chronic pain. Application. The review concludes by examining neuroimaging studies of neurodevelopmental and neurological disorders such as autism spectrum disorder (ASD), attention deficit hyperactivity disorder (ADHD), Alzheimer’s disease (AD), and Parkinson’s disease (PD). We also described the role of transcranial direct current stimulation (tDCS) in ASD, ADHD, AD, and PD. Neuroimaging techniques have significantly advanced our understanding of brain function and provided essential insights into neurological disorders. However, further research into noninvasive treatments such as EEG, MRI, and TES is necessary to continue to develop new diagnostic and therapeutic strategies for neurological disorders.

## 1. Introduction

Neuroimaging plays a crucial role in studying brain functions as it allows us to non-invasively observe the structure and activity of the brain [1,2]. By visualizing the brain’s structure and activity, neuroimaging techniques can help us understand how different brain regions are involved in various cognitive and behavioral processes, such as perception, attention, memory, language, decision-making, and emotion regulation [3]. Some standard neuroimaging techniques used in cognitive neuroscience research include fMRI, positron emission tomography (PET), EEG, and magnetoencephalography (MEG) [4,5]. These techniques can provide researchers with different spatial and temporal resolution levels, allowing them to investigate brain activity at different scales of time and space. For example, fMRI measures changes in blood flow in the brain, which reflect changes in neural activity. This technique can localize brain activity to specific regions and has been used to study various cognitive processes, such as attention and working memory. On the other hand, PET measures changes in the brain’s metabolism and can provide information about the brain’s neurochemical activity. EEG and MEG measure the electrical and magnetic activity generated by neural activity, respectively, and can provide information about the brain’s temporal dynamics. Neuroimaging is crucial in studying brain function, especially in severe psychiatric disorders—the study of people with bipolar disorder performing an emotional task while undergoing fMRI [6]. To check the effects of drug therapy for alcohol use disorder on the brain, researchers systematically reviewed fMRI, PET, single photon emission computed tomography (SPECT), and proton magnetic resonance spectroscopy (H-MRS) to assess treatment outcomes using neuroimaging techniques [7].

A comprehensive overview of the importance and impact of neuroimaging in neuroscience. Implications of Neuroimaging: Neuroimaging allows researchers to visualize brain activity, map neural networks, and explore the mechanisms underlying various neurological disorders. Neuroimaging has revolutionized our ability to study cognition, emotion, perception, and other fundamental aspects of brain function by capturing the dynamic nature of brain processes. Impact on Neuroscience: We further elaborate on the impact of neuroimaging on neuroscience. Neuroimaging has facilitated breakthroughs in our understanding of brain organization, connectivity, and plasticity. It also opens new avenues for studying the neural correlates of behavioral, cognitive, and psychiatric disorders. We discuss how neuroimaging techniques can help bridge the gap between neural activity and mental processes, leading to significant advances in understanding brain-behavior relationships. Clinical Applications: In addition to discussing the impact of neuroimaging on basic neuroscience research, we also highlight its critical role in clinical applications. Neuroimaging has revolutionized the diagnosis and treatment of neurological and psychiatric disorders. It enables the identification of biomarkers, assessment of treatment response, and development of personalized therapies. We highlight the contribution of neuroimaging to the study of neurodevelopmental disorders, neurodegenerative diseases, and psychiatric disorders. Overall, neuroimaging techniques have revolutionized our understanding of the brain and contributed significantly to cognitive neuroscience development. By allowing us to observe the brain’s activity and structure, neuroimaging has enabled researchers to test theories of brain function and develop new hypotheses about the neural basis of cognition and behavior.

According to the content of the Review, the following are some relevant keywords that can be used to conduct a literature search related to neuroimaging and its applications: neuroimaging, brain function, neurological diseases, fMRI, EEG, MRI, brain activity, neuro concussion, DTI, TES, Brain Connectivity, White Matter Tracts, Schizophrenia, Chronic Pain, ASD, ADHD, AD, PD, tDCS, Diagnostic Strategies, and Treatment Strategies. When conducting a literature search, describe the databases and sources searched, e.g., PubMed, Scopus or other relevant platforms that provide information on search terms and keywords used to identify relevant research. These keywords can be combined with Boolean operators such as “AND”, “OR” and “NOT” to refine the search and find relevant articles. In addition, there are considerations for using specific terms related to topics of interest in neuroimaging, such as particular brain regions, cognitive processes, or specific methods and techniques.

## 2. Noninvasive Functional Neuroimaging Methods

The importance of fMRI and EEG in studying brain activity and neurological disorders. fMRI and EEG are the most widely used and well-established neuroimaging techniques. fMRI and EEG provide complementary information about brain activity. fMRI measures changes in blood oxygen levels, allowing brain activity to be assessed with excellent spatial resolution. In contrast, an EEG records the brain’s electrical activity with high temporal resolution. By focusing on these two techniques, we can better understand brain dynamics, integrating the spatial and temporal aspects of brain function. Furthermore, both fMRI and EEG are noninvasive and, therefore, safe and well-tolerated by participants. This noninvasiveness allows for repeated measures and longitudinal studies, which are critical for studying brain changes associated with neurological disorders and evaluating therapeutic interventions. fMRI and EEG have broad applications in studying brain function and disease. They have been used to study various cognitive processes, emotional responses, sensory perception, and motor function. In addition, these techniques help characterize the brain in neurological disorders such as autism spectrum disorder (ASD), attention deficit hyperactivity disorder (ADHD), Alzheimer’s disease (AD), and Parkinson’s disease (PD)—changes in activity and connectivity. In conclusion, fMRI and EEG were chosen as the focus of our review because of their widespread use, complementary information, noninvasiveness, wide range of applications, and importance in studying brain activity and neurological disorders.

### 2.1. fMRI

#### 2.1.1. Recent Advances in fMRI Technology

fMRI technology has seen several recent advances, including Higher spatial resolution: The spatial resolution has increased over the years, with some scanners now capable of imaging voxels as small as 1 mm [8]. This increase in spatial resolution allows researchers to study smaller brain structures and activity patterns. Real-time fMRI: Real-time fMRI enables researchers to observe brain activity in real-time, allowing for more immediate feedback during experiments [9,10,11]. This technology can potentially be used in therapeutic interventions such as neurofeedback. Multimodal imaging: Combining fMRI with other imaging techniques, such as EEG and MEG, can provide a more comprehensive understanding of brain activity, allowing researchers to study both the brain function’s spatial and temporal dynamics [12,13]. Resting-state fMRI: Resting-state fMRI is a technique that measures brain activity while a person is at rest, not performing any specific task [14]. This technique has shown promise in identifying functional connectivity between brain regions and detecting changes in brain activity due to neurological disorders. Ultra-high field fMRI: Ultra-high field fMRI scanners, operating at 7 Tesla or higher, provide increased sensitivity to brain activity and higher spatial resolution [15].

#### 2.1.2. Applications of fMRI in Studying Brain Function

fMRI has a wide range of applications in studying brain function. Some of the main applications include mapping brain activity: fMRI is often used to identify regions of the brain that are active during specific tasks or stimuli [4]. This allows researchers to map functional networks and gain insights into how different brain regions are connected and work together. Understanding cognitive processes: Using fMRI to study brain activity during specific cognitive tasks, researchers can gain insights into how the brain processes information related to perception, attention, memory, language, decision-making, and other cognitive processes [16]. Recent advances in fMRI technology have significantly advanced our understanding of brain function and disease. The following are some notable advances: Multiband fMRI: Multiband imaging accelerates data acquisition and improves the temporal resolution of fMRI by acquiring multiple slices simultaneously. This advance enables the study of fast brain dynamics and real-time functional connectivity. Functional connectivity: Advances in fMRI connectivity analysis have facilitated the characterization of functional connectivity between different brain regions or networks. Seed-based correlations, independent component analysis (ICA), and graph theory have been used to map and study available connectivity patterns associated with various cognitive processes and neurological disorders. Multimodal Imaging Integration: Combining fMRI with other imaging modalities, such as EEG and MEG, provides a complete understanding of brain function. These multimodal approaches can simultaneously measure neural activity at different spatial and temporal scales, providing complementary information and enhancing the interpretation of fMRI findings. These recent advances in fMRI techniques, imaging methods, and data analysis techniques have greatly expanded our understanding of brain function and disease. They provide new avenues for studying functional and structural connectivity, mapping brain networks, decoding cognitive processes, and identifying neurological disease biomarkers. Studying neurological disorders: fMRI is often used to study changes in brain function associated with neurological disorders such as AD, PD, schizophrenia, and depression [17,18]. This can provide insights into the underlying mechanisms of these disorders and help with diagnosis and treatment. Developing new therapies: By using fMRI to identify regions of the brain associated with specific disorders, researchers can develop new therapies that target these regions [19]. For example, fMRI-guided deep brain stimulation has been used to treat PD and depression. Overall, fMRI is a powerful tool that can provide insights into the functioning of the human brain and has a wide range of applications in both basic and applied research.

#### 2.1.3. Recent Advances in Neuroimaging Techniques and Their Impact on Neuroscience Research

Recent advances in neuroimaging techniques have enabled researchers to investigate the brain’s structure and function with greater precision and detail. Here are some recent advances in neuroimaging techniques and their impact on neuroscience research: High-resolution structural MRI: MRI technology has enabled researchers to obtain high-resolution images of the brain’s structure, including the cortex’s sub-millimeter structures [20]. This has allowed for more accurate brain mapping and identifying previously unknown brain regions. Diffusion-weighted MRI: Diffusion-weighted MRI (DW-MRI) is a technique that measures the movement of water molecules in the brain’s white matter tracts [21]. This technique has enabled researchers to map the brain’s white matter connections and investigate how changes in these connections relate to brain disorders. Resting-state fMRI: Resting-state fMRI (rs-fMRI) is a technique that measures the brain’s intrinsic activity while the person is at rest [22]. This has allowed researchers to investigate the brain’s functional networks and their relationship to cognitive and behavioral processes. Multimodal neuroimaging: Multimodal neuroimaging involves combining two or more neuroimaging techniques, such as fMRI and EEG or fMRI and PET, to obtain complementary information about brain structure and function [23,24]. This has allowed for more comprehensive investigations of the brain’s neural activity. Ultrafast fMRI: Ultrafast fMRI techniques, such as multiband fMRI, have enabled researchers to obtain images of the brain’s activity faster, allowing for more accurate measurements of dynamic changes in neural activity [25]. Recent advances in neuroimaging techniques have expanded the field of cognitive neuroscience, providing researchers with new tools to investigate the brain’s structure and function. These techniques have led to a better understanding of brain disorders and have the potential to inform the development of new treatments and therapies.

#### 2.1.4. fMRI Data Analysis Methods or Algorithms

Several specific fMRI data analysis methods and algorithms are used to study brain function and neurological diseases. Seed-based functional connectivity: This method involves selecting a seed region of interest and computing correlations between the time series of the seed region and all other brain voxels. It allows researchers to study the functional connectivity between seed regions and the rest of the brain, revealing networks and connectivity patterns associated with specific brain functions or diseases. Independent Component Analysis (ICA): ICA is a data-driven method that decomposes fMRI data into spatially independent components representing distinct functional networks. It can identify resting-state networks, such as the default mode network, and examine their changes in various diseases. Graph-Theoretic Analysis: Graph theory provides a framework for describing and quantifying the topological properties of brain networks derived from fMRI data. Metrics such as node degree, clustering coefficient, and betweenness centrality can be used to assess network organization and identify network-level changes associated with brain disease. Machine Learning Methods: Machine learning algorithms have been applied to fMRI data to classify brain states, predict clinical outcomes, and identify biomarkers. Techniques such as support vector machines (SVM), random forests, and deep learning have extracted meaningful patterns and features from fMRI data, enabling accurate classification and prediction of various neurological disorders. Multivariate Pattern Analysis (MVPA): MVPA involves training machine learning algorithms on fMRI data to decode or differentiate between different mental states or stimuli. It allows researchers to study fine-grained patterns of brain activity associated with specific cognitive processes or conditions. It has been used in various fields, including cognitive neuroscience and clinical research. The area of fMRI data analysis is constantly evolving, with researchers exploring innovative ways to extract meaningful information from complex brain imaging data and improve our understanding of brain function and neurological disorders.

#### 2.1.5. Recent Advancements in fMRI Technology Improved the Spatial and Temporal Resolution

Recent advances in fMRI technology have significantly improved spatial and temporal resolution, thereby increasing the accuracy and specificity of brain activity measurements. Here are some critical advances and their implications: High-field MRI: Using higher magnetic field strengths, such as 3 Tesla (3T) or even 7 Tesla (7T), improves the spatial resolution of fMRI. Higher field strengths can better distinguish small brain structures and finer details, allowing researchers to detect and localize brain activity more precisely. Multi-echo Imaging: A multi-echo fMRI sequence acquires multiple echoes at different time points, allowing various aspects of the fMRI signal to be captured. By combining these echoes, researchers can separate the BOLD (blood oxygen level dependent) signal from other confounding sources of signal variation, improving the specificity of measuring neural activity. Parallel Imaging: Parallel imaging techniques, such as Sensitivity Encoding (SENSE) and Generalized Autocalibration Partially Parallel Acquisition (GRAPPA), utilize multiple receive coils to speed up image acquisition and improve temporal resolution. The faster acquisition allows dynamic changes in brain activity to be captured with higher temporal fidelity. Simultaneous Multi-Slice (SMS) Imaging: SMS imaging technology acquires multiple slices of the brain simultaneously, increasing coverage and reducing acquisition time. This enables faster whole-brain imaging with higher temporal resolution, which is particularly beneficial for studying rapidly evolving brain processes. Ultrafast Echo Planar Imaging (EPI): EPI is the most commonly used fMRI acquisition method, and recent advances have focused on improving its speed and image quality. Advanced EPI techniques, such as multiband imaging and blipped-CAIPI, have significantly reduced acquisition times while maintaining acceptable image quality, enabling higher temporal resolution and improved detection of fast neural events.

### 2.2. EEG

#### 2.2.1. Recent Advances in EEG Technology

EEG is a non-invasive technique for measuring the brain’s electrical activity [26,27,28,29]. Recent advances in EEG technology include High-density electrode arrays: EEG systems with high-density electrode arrays, consisting of hundreds of electrodes, have become increasingly common in recent years [30]. These systems allow for more precise localization of brain activity and can better capture the dynamics of neural networks. Real-time source localization: Real-time source localization is a new technology that identifies the location and strength of brain activity in real-time [31]. This is particularly useful for applications such as neurofeedback and brain-computer interfaces, which require real-time analysis of brain activity. Wearable EEG technology: Recent developments in wearable EEG technology have made it possible to measure brain activity outside of traditional laboratory settings [32]. This has potential applications in clinical settings, where it may be possible to use wearable EEG to monitor brain activity in patients with neurological disorders. Integration with other neuroimaging techniques: EEG can be integrated with other neuroimaging techniques, such as fMRI, to provide complementary information about brain function [33,34]. For example, simultaneous EEG-fMRI can provide information about both brain activity’s spatial and temporal characteristics. Overall, these advances in EEG technology are improving our ability to study brain function and have potential applications in both basic and applied research.

#### 2.2.2. Applications of EEG in Studying Brain Function and Neural Oscillations

EEG is a powerful tool for studying brain function and neural oscillations [35]. Some of the main applications of EEG include mapping brain activity: EEG can be used to map the location and timing of brain activity associated with different cognitive processes. This can provide insights into the neural mechanisms underlying these processes. Studying neural oscillations: EEG can be used to study the rhythmic patterns of neural activity, known as neural oscillations [36]. These oscillations are thought to play a critical role in information processing in the brain and can be studied using advanced analytical techniques such as spectral analysis and event-related potentials. Investigating brain connectivity: EEG can be used to investigate the connectivity between different brain regions, both in terms of functional connectivity and effective connectivity (the causal relationships between brain regions) [37,38]. This can provide insights into the neural networks that underlie cognition and behavior. Studying brain development: EEG can be used to study changes in brain function and neural oscillations during brain development [39]. This can provide insights into the development of cognitive functions such as language, attention, and memory. EEG can also be used to map the location of brain activity and identify areas of the brain that are critical for motor, language, and other functions. Overall, EEG is a versatile and valuable tool for studying brain function and neural oscillations.

EEG signal processing techniques are crucial in extracting meaningful information about brain activity and analyzing neural oscillations. Preprocessing: Preprocessing is an essential step in removing artifacts and improving the quality of EEG data. It includes filtering to remove noise (e.g., power line interference, muscle artifacts), artifact removal techniques (e.g., independent component analysis, template matching), and baseline correction to establish a reference point for further research. Power Spectrum Analysis: Power Spectrum Analysis examines power distribution in different frequency bands in the EEG signal. The Fast Fourier Transform (FFT) is commonly used to compute the power spectrum, which provides information about the magnitude of neural oscillations in a specific frequency range (e.g., delta, theta, alpha, beta, gamma). Power spectral analysis can reveal changes in oscillatory activity associated with different cognitive states or pathologies. Event-Related Potential (ERP): An ERP is a transient voltage change in an EEG signal that is time-locked to a specific event or stimulus. ERP analysis involves averaging EEG segments aligned with event onset to improve the signal-to-noise ratio. This technique enables researchers to study brain responses to stimuli or cognitive processes with high temporal precision and to identify components such as P300, N400, or sensory evoked potentials. Time-Frequency Analysis: Time-frequency analysis provides information about the spectral content of the EEG signal over time. Techniques such as the short-time Fourier transform (STFT), wavelet transform, or Hilbert transform can compute time-frequency representations such as spectrograms or time-frequency plots. This analysis enables researchers to examine oscillatory activity changes over time and identify transient or task-specific spectral modulations. Connectivity analysis: Connectivity analysis aims to study the functional connectivity between brain regions based on EEG data. Measures such as coherence, phase synchrony, or Granger causality can be used to quantify the strength and directionality of functional connectivity. Connectivity analysis helps reveal network dynamics and interactions between brain regions, providing insight into information flow and integration in the brain. Source Localization: Source localization techniques estimate potential neural sources responsible for recorded EEG signals. Various methods, including dipole fitting, distributed source modeling (e.g., standardized low-resolution electromagnetic tomography of the brain—sLORETA), and beamforming, can localize activity to specific brain regions or cortical sources. Source localization helps identify the neural generators of EEG signals and allows for more precise brain activity mapping.

#### 2.2.3. Some Recent Developments in EEG Technology have Enhanced Its Utility in Studying Brain Activity and Disease

##### High-Density EEG and Advanced Electrode Design

High-density EEG: Traditional EEG systems use a limited number of electrodes, resulting in sparse spatial scalp coverage. However, recent advances have led to high-density EEG systems with more electrodes (e.g., 128, 256, or even 512 channels). High-density EEG allows for more precise spatial mapping of brain activity and improved localization of neural sources. Flexible Dry Electrodes: Conventional EEG electrodes require conductive gel or paste to establish good electrical contact with the scalp. In contrast, loose and dry electrodes have been developed, which offer more comfortable and user-friendly options for electrode placement. In addition, these electrodes can maintain good signal quality and reduce preparation time, making them more suitable for long-term monitoring and clinical applications.

##### Signal Processing and Analysis Techniques: Source Localization

Advanced source localization algorithms have been developed to estimate the location of neural sources under EEG signals. These techniques utilize high-density electrode configurations and sophisticated mathematical modeling to increase the accuracy and resolution of source localization. Connectivity analysis: Like fMRI, EEG data can be analyzed to study functional connectivity patterns between brain regions. Connectivity analysis techniques, such as coherence, phase synchrony, and graph theory, have been applied to EEG data to understand coordination and communication between brain regions.

##### Integration with Other Modalities

fMRI-EEG fusion: Combining EEG with fMRI allows simultaneous EEG and hemodynamic activity measurement, providing complementary information about brain function. fMRI-EEG fusion techniques enable researchers to study the relationship between neural oscillations measured by EEG and underlying brain networks identified by fMRI. EEG-TMS Integration: Transcranial Magnetic Stimulation (TMS) is a non-invasive brain stimulation technique that modulates cortical excitability. EEG-TMS integration enables researchers to measure the direct effects of TMS on brain activity and investigate the mechanisms underlying the therapeutic and cognitive impacts of TMS interventions.

##### Wearable and Mobile EEG

Advances in miniaturization and wireless technology have led to the development of wearable and mobile EEG devices. These devices are lightweight, portable, and comfortable to wear, allowing for more natural and ecologically valid brain activity measurements in various environments and real-life scenarios.

These recent developments in EEG technology have expanded its utility for studying brain activity and disease. Advances in electrode design, signal processing algorithms, and integration with other modalities have increased spatial resolution, signal quality, and the ability to study neural dynamics and connectivity patterns. These advances can potentially enhance the diagnosis, monitoring, and treatment of various neurological and psychiatric disorders.

### 2.3. The Limitations and Challenges Associated with fMRI and EEG Techniques

Limitations and challenges associated with fMRI and EEG techniques to provide a balanced perspective on their applicability and reliability. The following are some of the main rules and challenges of fMRI and EEG:

#### 2.3.1. Spatial Resolution

While fMRI offers excellent spatial resolution, it is still limited in resolving fine-scale neural activity. Spatial resolution is typically on the order of millimeters and may not accurately capture the action at the level of individual neurons or cerebellar regions. EEG has poor spatial resolution. The electrical activity recorded by EEG electrodes is an aggregated signal from a large amount of brain tissue, making pinpointing the source of the exercise challenging.

#### 2.3.2. Temporal Resolution

fMRI Despite its high spatial resolution, the temporal resolution of fMRI is relatively poor. The blood oxygen level-dependent (BOLD) signal measured in fMRI reflects hemodynamic changes that occur with a delay of several seconds, limiting its ability to capture fast neural dynamics. EEG excels in temporal resolution, capturing millisecond-level brain activity. However, it may not accurately detect slower, longer-lasting neural processes.

#### 2.3.3. Signal-to-Noise Ratio (SNR)

fMRI signals are susceptible to noise from various sources, such as motion artifacts, physiological fluctuations, and scanner-related distortions. These noise sources reduce the signal-to-noise ratio, affecting fMRI measurements’ reliability. In addition, EEG signals are relatively weak and can be affected by various artifacts, including muscle activity, eye movements, and ambient electrical noise. These artifacts can degrade the quality of the EEG recording and influence the interpretation of the data.

#### 2.3.4. Interpretation Challenges

Interpreting fMRI results requires careful consideration of the complex relationship between the BOLD signal and the underlying neural activity. The BOLD signal represents an indirect measure of neural activity and is influenced by vascular and metabolic factors, which may limit the specificity of findings. Analyzing EEG data involves addressing challenges associated with source localization and isolating overlapping neural sources. In addition, EEG signals are subject to volumetric conduction effects, making it difficult to attribute specific activity to particular brain regions.

Despite these limitations and challenges, fMRI and EEG remain invaluable tools for studying brain activity and neurological disorders. However, with careful consideration and appropriate methodology, fMRI and EEG techniques provide valuable insights into brain function and significantly contribute to our understanding of neurological disorders.

## 3. Advanced Techniques in Neuroimaging

### 3.1. DTI

#### 3.1.1. Recent Advances in DTI Technology

DTI is a type of MRI used to visualize the white matter tracts in the brain [40]. Recent advances in DTI technology include High angular resolution diffusion imaging (HARDI): HARDI is a variant of DTI that allows for more detailed visualization of complex white matter structures [41]. It uses better angular resolution to capture the directionality of water diffusion in brain tissue, resulting in more accurate tractography and more detailed maps of white matter connectivity. Accelerated imaging: New techniques for accelerated DTI imaging, such as compressed sensing and parallel imaging, can significantly reduce scan times while maintaining image quality [42]. This is particularly important for clinical applications, where shorter scan times can improve patient comfort and reduce motion artifacts. These advances in DTI technology are improving our ability to study the structure and connectivity of white matter in the brain and have potential applications in clinical diagnosis and the treatment of neurological disorders.

#### 3.1.2. Applications of DTI in Studying Brain Connectivity and White Matter Tracts

DTI is a powerful tool for studying brain connectivity and white matter tracts [43]. Some of the main applications of DTI include: Mapping white matter tracts: DTI is often used to visualize and map white matter tracts in the brain [44]. This allows researchers to study the connectivity between different brain regions and to investigate the relationship between brain structure and function. Identifying structural changes in neurological disorders: DTI can be used to identify changes in white matter structure and connectivity associated with neurological disorders such as AD, multiple sclerosis, and traumatic brain injury [45,46,47]. This can help diagnose, monitor disease progression, and develop new treatments. Studying brain development: DTI can be used to study changes in white matter structure and connectivity during brain development [43]. This can provide insights into the development of cognitive functions such as language, attention, and memory. Investigating brain plasticity: DTI can investigate changes in white matter structure and connectivity associated with learning and training [48]. This can provide insights into the neural mechanisms of brain plasticity and can have potential applications in rehabilitation.

Here is how DTI can help us understand brain connections and white matter tracts: Mapping white matter tracts: DTI can reconstruct and visualize major white matter tracts in the brain, such as the corpus callosum, corticospinal tract, and cingulate tract. By estimating the predominant diffusion direction in each voxel, DTI generates fiber orientation maps that can be used to track the trajectories of white matter tracts and study their connectivity patterns. Quantifying Diffusion Indexes: DTI provides several diffusion indices that characterize white matter microstructure. The most commonly used metric is fractional anisotropy, which reflects water diffusion’s directionality and degree of coherence within a voxel. Other metrics include mean diffusivity, axial diffusivity, and radial diffusivity. These metrics provide quantitative measures of white matter integrity, axonal density, and myelination which may be associated with specific neurological disorders or brain development. Studying brain connectivity: DTI-based tractography allows researchers to explore structural connections between different brain regions. By reconstructing fiber pathways, DTI enables the investigation of long-range connections and the mapping brain networks. Combined with functional connectivity analysis that examines the temporal correlation of brain activity, DTI helps reveal the relationship between structural and functional connectivity, providing insights into brain organization and communication. Recent advances in DTI analysis methods aim to improve their accuracy and efficiency. Some notable advances include High Angular Resolution Diffusion Imaging (HARDI): Compared to traditional DTI, HARDI techniques, such as Diffusion Spectral Imaging and High Definition Fiber Tracking, provide more detailed information on complex fiber orientations. These methods allow for better characterization of intersecting fibers, leading to more accurate reconstruction of white matter tracts and improved delineation of connectivity patterns. Multi-shell and multi-tissue modeling: Multi-shell diffusion acquisition combined with advanced modeling methods allows the estimation of diffusion parameters across multiple compartments within each voxel. This enables the separation of isotropic diffusion from anisotropic diffusion, providing more specific information about tissue microstructure and improving the accuracy of tractography algorithms. Advanced tractography algorithms: Various advanced tractography algorithms have been developed to improve the accuracy and reliability of DTI-based fiber tracking. These algorithms incorporate more sophisticated techniques such as probabilistic modeling, constrained spherical deconvolution, and anatomical priors to increase tractography results’ robustness and spatial specificity. These advances in DTI analysis methods have improved the accuracy, efficiency, and interpretability of DTI data, enhancing our understanding of brain connectivity and white matter tracts. They enable more precise mapping of fiber pathways, better quantification of microstructural properties, and more complex studies of brain networks, ultimately improving our understanding of brain structure and function in health and disease.

### 3.2. Transcranial Electrical Stimulation (TES)

#### 3.2.1. Recent Advances in TES Technology

TES is a non-invasive brain stimulation technique that involves applying low-intensity electrical currents to the scalp to modulate brain activity [49,50,51]. In recent years, several advances in TES technology have improved its effectiveness and expanded its potential applications. Here are some recent advances in TES technology: High-Definition Transcranial Direct Current Stimulation (HD-tDCS): HD-tDCS is a newer form of tDCS that uses a more focused electrical field to stimulate specific regions of the brain [52]. This technique uses smaller electrodes with more contact points, allowing for more precise targeting of brain areas. As a result, HD-tDCS is more effective than traditional tDCS in improving cognitive and motor functions. This technique has been shown to improve cognitive functions such as attention, memory, and learning. Personalized TES involves using individualized brain mapping techniques to target specific brain regions for stimulation [53]. This approach has been shown to improve the effectiveness of TES in treating conditions such as depression, schizophrenia, and chronic pain. Combined TES and Neuroimaging: Combining TES with neuroimaging techniques such as EEG and fMRI allows researchers to understand better the mechanisms underlying TES and to optimize stimulation protocols for individual patients [54,55]. Recent TES technology advances have expanded its potential applications and improved its effectiveness as a non-invasive brain stimulation technique.

#### 3.2.2. Applications of TES in Stimulating the Brain and as a Potential Treatment for Schizophrenia and Chronic Pain

TES has been studied as a potential treatment for various neurological and psychiatric disorders, including schizophrenia and chronic pain [56]. Here are some of the applications of TES in stimulating the brain and as a potential treatment for schizophrenia and chronic pain: Schizophrenia: TES has been studied as a potential treatment for schizophrenia, a chronic mental disorder characterized by symptoms such as delusions, hallucinations, and disordered thinking [57]. Several studies have shown that TES can improve symptoms of schizophrenia, including auditory hallucinations and negative symptoms. TES techniques such as tDCS and transcranial magnetic stimulation (TMS) have been used to stimulate specific brain regions. Chronic pain: TES has also been studied as a potential treatment for chronic pain, a common and often debilitating condition affecting millions worldwide [58]. TES techniques such as tDCS and transcranial alternating current stimulation (tACS) effectively reduce chronic pain in various conditions, including fibromyalgia, neuropathic pain, and migraine headaches [59]. In addition, TES can modulate the activity of pain pathways in the brain, reducing pain perception. Motor functions: TES has been studied as a potential treatment for motor impairments, including stroke and Parkinson’s disease [60,61]. Cognitive functions: TES has also been studied as a potential treatment for cognitive impairments, including memory and attention deficits [62,63]. TES can improve cognitive functions by modulating the activity of specific brain regions involved in these functions, such as the prefrontal cortex. TES has shown promise as a non-invasive and potentially effective treatment for various neurological and psychiatric disorders.

TES techniques, including tDCS, have been investigated as potential treatments for various neurological and psychiatric disorders, including schizophrenia and chronic pain. Here are some examples of research and findings in these areas: tDCS in Schizophrenia: Brunelin et al. (2012): This study examined the effect of tDCS on auditory hallucinations in patients with schizophrenia. They found that active tDCS applied to the left temporoparietal junction significantly reduced the severity and frequency of auditory hallucinations compared with sham stimulation [64]. Mondino et al. (2016): In this study, tDCS was applied to the prefrontal cortex of patients with schizophrenia. Results showed improvements in cognitive functions, such as working memory and attention, suggesting a potential role for tDCS in enhancing cognitive deficits associated with schizophrenia [65]. tDCS in chronic pain: Straudi et al. (2018): This randomized controlled trial investigated the effects of tDCS in patients with chronic nonspecific low back pain. The study showed that active tDCS applied to the motor cortex significantly reduced pain intensity compared to sham stimulation [66]. Luedtke et al. (2015): This study used tDCS on patients with fibromyalgia, a chronic pain disorder. It was shown that active tDCS applied to the primary motor cortex significantly reduced pain intensity and improved other fibromyalgia-related symptoms compared with sham stimulation [67]. These studies highlight the potential of TES techniques such as tDCS as non-invasive interventions for schizophrenia and chronic pain. While these findings are promising, it is essential to note that further research is needed to determine the efficacy, optimal stimulation parameters, and long-term effects of TES under these conditions.

### 3.3. DTI and TES for Understanding Brain Connections and Researching Treatments for Disorders Such as Schizophrenia and Chronic Pain

#### 3.3.1. Applications of DTI: Mapping White Matter Tracts

DTI allows researchers to map the trajectories of major white matter tracts in the brain, providing insight into the anatomical connections between different brain regions. Studying brain connectivity: By quantifying the direction and magnitude of water diffusion, DTI can estimate diffusion tensor metrics such as fractional anisotropy (FA) and mean diffusivity (MD). These metrics can be used to study the integrity and connectivity of white matter pathways. Understanding Neurological Disease: DTI has been used extensively to study altered white matter connectivity in various neurological and psychiatric disorders, including schizophrenia, Alzheimer’s disease, multiple sclerosis, and traumatic brain injury. It helps identify specific white matter abnormalities associated with these disorders and understand their impact on brain function. High Angular Resolution Diffuse Imaging (HARDI): HARDI techniques, such as q-sphere and diffuse spectral imaging, improve DTI accuracy and resolution by modeling complex fiber orientations and overcoming limitations associated with intersecting fibers. Tractor imaging and connectivity analysis: Advanced tractography algorithms have been developed to reconstruct white matter pathways more accurately. With connectivity analysis techniques like graph theory, DTI allows studying structural brain networks and their alterations in neurological diseases.

#### 3.3.2. Applications of TES: Non-Invasive Brain Stimulation

TES techniques, such as transcranial direct current stimulation (tDCS) and transcranial alternating current stimulation (tACS), provide non-invasive methods to modulate brain activity and study the functional connectivity of brain networks. Investigating brain function: TES can probe causal relationships between brain regions and explore the active contribution of specific brain networks. In addition, it allows researchers to study the effects of targeted brain stimulation on behavior, cognition, and perception. Potential Therapeutic Applications: TES has shown promise as a treatment option for various neurological and psychiatric disorders. For example, Schizophrenia: Studies have explored using TES, particularly tDCS, as an adjunctive treatment for schizophrenia. By modulating cortical excitability and network connectivity, tDCS has the potential to reduce symptoms and improve cognitive function in patients with schizophrenia. Chronic pain: TES techniques, such as transcranial electrical nerve stimulation (TENS), have been used as non-invasive pain management strategies. By targeting specific pain-related brain regions, TES can modulate pain perception and relieve people with chronic pain. Targeted Stimulation: Advances in TES technology allow for more precise targeting of specific brain regions or networks. High-resolution neuroimaging techniques, such as fMRI or EEG, can be used with TES to guide electrode placement and optimize stimulation protocols.

### 3.4. Limitations, Challenges, Potential Future Directions, and Improvements Related to DTI and TES Techniques

#### 3.4.1. Limitations and Challenges of DTI

Tissue Complexity: DTI assumes a simplified water diffusion model in biological tissue, which may not fully capture the complex microstructural properties of brain tissue. This can present challenges in accurately interpreting and quantifying diffusion indicators. Fiber crossings and orientation dispersion: DTI strives to resolve complex fiber configurations, such as regions with fiber crossings or orientation dispersion. This limitation leads to inaccurate white matter tract reconstruction and connectivity estimates. Sensitivity to noise and artifacts: DTI is sensitive to noise and various artifacts, such as motion artifacts and distortions caused by susceptibility, which degrade image quality and affect the reliability of diffusion indicators. Spatial resolution: High spatial resolution in DTI is challenging due to long acquisition times and susceptibility to image distortion, limiting the ability to resolve small-scale white matter structures.

#### 3.4.2. Future Directions and Improvements for DTI

Advanced diffusion models: Developing more advanced diffusion models beyond simple tensor models can better represent tissue microstructure. High Angular Resolution Diffusion Imaging (HARDI) and Diffusion Kurtosis Imaging (DKI) are designed to capture complex fiber orientation and diffusion patterns. Multimodal integration: Integrating DTI with other imaging modalities, such as functional MRI (fMRI) or EEG, allows a complete understanding of the relationship between structural connectivity and available brain networks. Enhanced image acquisition: Improvements in acquisition protocols, such as higher field strengths, parallel imaging techniques, and motion correction methods, can help address challenges related to spatial resolution, image quality, and artifacts. Advanced Analytical Methods: Developing more sophisticated analytical techniques, such as advanced tractography algorithms and network-based methods, could improve the accuracy and robustness of white matter tractography reconstruction and connectivity estimation.

#### 3.4.3. Limitations and Challenges of TES

Spatial Localization and Individual Differences: Due to anatomical variation between individuals, pinpointing specific brain regions in TES can be challenging. Variations in skull conductivity and electrode placement affect the distribution and magnitude of the electric field. Limited Mechanistic Understanding: While TES has shown beneficial effects in various applications, the exact underlying mechanisms are not yet fully understood. Further studies are needed to elucidate the neurobiological impact of TES and optimize stimulation parameters. Inter- and within-subject variability: There is considerable inter- and within-subject variability in response to TES, making it challenging to establish standardized protocols and predict individual treatment outcomes. Potential side effects: While TES is generally considered safe, there may be possible side effects such as scalp irritation or mild discomfort during stimulation. The long-term effects of repeated or prolonged TES therapy are still being studied.

#### 3.4.4. Future Directions and Improvements in TES

Personalized approaches: Advances in neuroimaging techniques, such as personalized brain mapping using structural or functional connectivity data, can help guide personalized TES protocols and optimize electrode placement. Closed-loop and Adaptive Stimulation: Closed-loop TES systems that integrate real-time monitoring of brain activity and adjust stimulation parameters based on ongoing neural states hold the promise of personalized and optimized stimulation protocols. Multimodal Integration: Combining TES with neuroimaging techniques such as fMRI or EEG can provide real-time brain activity feedback and improve stimulation’s accuracy and targeting. Improved Electrode Design: Development of a more advanced and flexible electrode design to conform to the scalp, achieve reliable electrical contact, and minimize discomfort for improved efficacy.

By addressing these limitations and exploring future directions, both DTI and TES techniques can significantly improve our understanding of brain connectivity, neurodevelopmental disorders, and therapeutic interventions for various neurological and psychiatric disorders.

## 4. Neuroimaging and Brain Functions

### 4.1. Neuroimaging Studies on Neurodevelopmental Disorders, Such as ASD and ADHD

ASD is characterized by a range of symptoms and severity, which is why it is called a “spectrum” disorder. Research has shown that people with autism have abnormalities in brain structure and function, especially in areas responsible for communication, social interaction, and sensory processing [68,69]. These abnormalities can cause disruptions in neural networks critical to learning, memory, and emotion regulation. ASD is more common in boys than girls, affecting about 1 in 54 children in the United States [70]. The pathology of ASD involves a spectrum of symptoms that affect communication, social interaction, and behavior. The exact cause of ASD is unknown, and there is no known cure. Neuroimaging studies have provided valuable insights into the brain mechanisms underlying neurodevelopmental disorders such as ASD [71,72,73]. Here are some brief examples of what has been discovered in these areas through neuroimaging research in ASD [74,75,76]. For example, studies have found differences in the size and shape of the amygdala, a brain region involved in emotional processing [77,78]. Other studies have found differences in the structure and connectivity of the prefrontal cortex, which is involved in social cognition and executive functioning [79,80].

ADHD is one of the most common neurodevelopmental disorders, affecting approximately 6–9% of children and 5% of adults worldwide [81,82]. Studies have shown differences in the structure and function of certain parts of the brain in people with ADHD compared to people without the disorder [83]. Specifically, the study found abnormalities in the prefrontal cortex, basal ganglia, and cerebellum involved in attention, motivation, and motor control [84,85]. The pathology of ADHD consists of a spectrum of symptoms that affect an individual’s ability to pay attention, control impulsive behavior, and regulate activity levels. These symptoms can vary in severity and interfere with an individual’s academic, social, and occupational functioning. For example, studies have found differences in the size and shape of the basal ganglia, a brain region involved in motor control and reward processing [86,87]. Other studies have found differences in the activity and connectivity of the prefrontal cortex, which is involved in attentional control and executive functioning [88,89]. Overall, neuroimaging studies have provided important insights into the brain mechanisms underlying neurodevelopmental disorders.

#### 4.1.1. TES in ASD

In recent years, TES has gained increasing attention as a potential therapeutic intervention for ASD [90,91,92,93,94,95,96,97,98]. One type of TES that has been studied in ASD is tDCS, which involves the application of a weak electrical current to the scalp to modulate cortical excitability. tDCS, a non-invasive brain stimulation neuromodulation technology, is a promising method for treating ASD [90,93,94,95,97,99]. The following is a summary in Table 1. Several studies have investigated the effects of tDCS in individuals with ASD, including changes in brain metabolism, long-term impact on symptoms, social cognition, and social skills, modulation of EEG activity, and effects on motor and balance skills. Some studies have reported positive results of tDCS. Overall, the current evidence suggests that tDCS may have potential as a treatment option for some symptoms of ASD.

#### 4.1.2. TES in ADHD

TES has been studied as a potential treatment for ADHD. One type of TES that has been studied in ADHD is tDCS, which involves the application of a weak electrical current to the scalp to modulate cortical excitability. Studies using tDCS have reported improved attention and hyperactivity in children with ADHD [100,101,102,103,104,105]. Therefore, several papers focus on the effects of tDCS on ADHD. The following is a summary in Table 2. Several studies have investigated the effects of tDCS on ADHD symptoms, including improvements in cognitive control, inhibitory control, attentional set-shifting tasks, and planning and working memory capacities. In addition, some studies have found significant improvements in ADHD symptoms following tDCS.

### 4.2. Neuroimaging Studies on Neurological Disorders, Such as AD and PD

AD is a progressive neurodegenerative disease that affects the brain and gradually impairs an individual’s memory, thinking, and behavior. It is the most common cause of dementia, accounting for 60–80% of cases [106]. These proteins form plaques and tangles that interfere with regular communication between brain cells and cause them to die [107,108,109]. The earliest symptoms of AD often involve memory loss, especially of recent events or information. As the disease progresses, individuals may experience difficulties with language, spatial awareness, problem-solving, and completing everyday tasks. They may also experience changes in mood, personality, and behavior [110]. A diagnosis of AD usually involves a comprehensive medical evaluation, including a neurological exam, cognitive tests, and imaging tests such as MRI scans [111]. Currently, there is no cure for AD, and treatment focuses on managing symptoms and slowing the progression of the disease. Neuroimaging studies have been instrumental in understanding the structural and functional changes that occur in the brains of individuals with neurological disorders such as AD [112,113]. Here are some brief examples of what has been discovered in these areas through neuroimaging research in AD. Neuroimaging studies have shown that individuals with AD have reduced brain volume, particularly in the hippocampus, which is critical for memory function [114,115]. They have also revealed changes in the connectivity between brain regions and alterations in glucose metabolism in specific brain regions [116,117].

PD is the second most common neurodegenerative disorder after Alzheimer’s and is estimated to affect about 1 percent of people over 60 [118]. The earliest signs of PD include tremors, stiffness, and slow movement. As the disease progresses, individuals may experience difficulty with balance, coordination, and fine motor skills [119]. In addition to motor symptoms, PD can cause non-motor symptoms such as depression, anxiety, and sleep disturbances. Therefore, diagnosing PD usually involves a comprehensive medical evaluation, including a neurological examination, imaging studies such as MRI scans, and evaluation of motor and non-motor symptoms [120]. Although PD cannot be cured, medications and other treatments can help manage symptoms and improve quality of life. Neuroimaging studies have shown that individuals with PD have reduced dopamine activity in the brain due to the degeneration of dopaminergic neurons [121,122]. They have also revealed changes in the size and connectivity of brain regions involved in movement control, such as the basal ganglia and motor cortex [123,124]. Overall, neuroimaging studies have been crucial in advancing our understanding of the brain mechanisms underlying neurological disorders. They have allowed for the detection of structural and functional changes in the brain that may aid in diagnosing and monitoring these disorders and may also help inform the development of new treatments.

#### 4.2.1. TES in AD

TES has been investigated as a potential treatment for AD. Some studies have reported improvements in cognitive function, memory, and daily living activities following TES with AD [125,126]. Studies using tDCS have reported improved cognitive function and memory in individuals with AD [127,128,129,130]. The following is a summary in Table 3. tDCS has been studied as a potential treatment for cognitive impairment and memory deficits in Alzheimer’s patients, and its positive effects on cognitive function and memory in AD patients. Overall, while the results of TES studies in AD are still preliminary, they suggest that it may be a promising therapeutic intervention for improving cognitive function and memory in individuals with AD.

#### 4.2.2. TES in PD

Some studies have reported improvements in motor symptoms, tremors, and gait following TES with PD [60,131]. One type of TES studied in PD is tDCS, which involves the application of a weak electrical current to the scalp to modulate cortical excitability. Studies using tDCS have reported improved motor symptoms, tremors, and gait in individuals with PD [132,133,134,135,136]. Several papers focus on the effects of tDCS on PD. The following is a summary in Table 4. Several studies have reported improved motor symptoms, tremors, and gait in individuals with PD using tDCS. Overall, while the results of TES studies in PD, there is some evidence to suggest that it may be a promising therapeutic intervention for improving motor symptoms and gait in individuals with PD.

### 4.3. tDCS in the Mentioned Neurodevelopmental and Neurological Disorders, Including Its Mechanisms of Action

Transcranial direct current stimulation (tDCS) is a non-invasive technique that uses a weak electrical current applied to the scalp to modulate neuronal activity in targeted brain regions. It involves placing an anode (positive electrode) and a cathode (negative electrode) at specific locations on the scalp, delivering a constant, low-intensity electrical current. The potential therapeutic application of tDCS in various neurodevelopmental and neurological disorders has been investigated. Here are insights into its specific role, mechanism of action, and supporting evidence in some of these diseases:

#### 4.3.1. ASD

The role of tDCS: tDCS has been explored as a potential intervention to improve social cognition, language skills, and executive function in patients with ASD. Mechanism of Action: tDCS modulates cortical excitability and enhances synaptic plasticity, resulting in functional changes in brain circuits associated with social communication and cognitive processes. Evidence: Several studies have shown that tDCS has favorable effects in improving social cognition, reducing repetitive behaviors, and enhancing language skills in individuals with autism. However, evidence remains limited and further research is needed to determine optimal stimulation parameters and long-term effects.

#### 4.3.2. ADHD

The role of tDCS: tDCS has been investigated as a potential nonpharmacologic intervention to enhance attention, working memory, and executive function in ADHD patients. Mechanism of Action: tDCS is thought to modulate neuronal excitability and restore abnormal brain activity patterns in attention and cognitive control areas. Evidence: Several studies have reported improvements in attention and cognition after tDCS in people with ADHD. However, study results are mixed, and more research is needed to determine the effectiveness of tDCS as a standalone or adjunctive treatment for ADHD.

#### 4.3.3. AD

The role of tDCS: tDCS has been explored as a potential intervention to enhance cognitive functions such as memory and attention in AD patients. Mechanism of Action: tDCS modulates cortical excitability and promotes neuroplasticity, potentially counteracting neuronal dysfunction and cognitive decline associated with AD—evidence: Several studies have shown that tDCS benefits cognition and memory in AD patients. However, evidence remains limited and further research is needed to determine optimal stimulation regimens, long-term effects, and potential synergies with other therapeutic interventions.

#### 4.3.4. PD

Role of tDCS: tDCS has been investigated as a potential adjunctive therapy to reduce motor symptoms and improve motor function in PD patients. Mechanism of Action: tDCS modulates cortical excitability and affects motor networks, potentially enhancing motor performance and reducing motor symptoms. Evidence: Several studies have reported improved motor function, gait, and motor symptoms after tDCS in PD patients. However, evidence remains limited, and more research is needed to determine optimal stimulation parameters, long-term effects, and potential synergy with other treatments.

It is important to note that while tDCS holds promise as a potential therapeutic tool for these diseases, the field is still actively exploring optimal stimulation parameters, individual differences in treatment response, and long-term effects of tDCS. Further, well-designed studies are needed to determine its efficacy, safety, and long-term benefits in neurodevelopmental and neurological disorders.

## 5. Summary of Recent Advances in Neuroimaging and Their Impact on Neuroscience Research and Clinical Practice

There are several reasons for choosing a narrative review over a systematic review: Scope and focus: A systematic review aims to comprehensively collect and synthesize all relevant published studies on a specific research question and follow a predefined methodology. However, the narrative review enabled the authors to provide a more focused and selective look at recent advances in noninvasive functional neuroimaging methods. It gives the flexibility to discuss critical studies, concepts, and trends without being constrained by systematic reviews. Time and resources: Conducting a systematic review requires significant time and resources. It involves rigorous search strategies, study screening and selection, data extraction, and statistical analysis. Systematic reviews can be time- and effort-intensive depending on the scope and breadth of the topic. Authors’ choice of narrative review may be based on constraints of time, resources, or available expertise. Purpose of the review: The authors’ aim may have been to provide a general overview of recent advances in noninvasive functional neuroimaging techniques rather than a comprehensive synthesis of all available evidence. They may prioritize discussions on the most relevant and influential critical studies, concepts, and trends. Although systematic reviews provide a more comprehensive analysis, narrative reviews can still offer value in summarizing and discussing recent advances in a field. Readers interpret and apply the results to their own research or clinical practice. In this case, one of the reasons for choosing a narrative review was to provide a quick and more comprehensive overview of the latest advances in noninvasive functional neuroimaging techniques. Narrative reviews are easier to conduct, can be completed in a shorter period, and may be more accessible to readers who introduce the topic.

**Figure 1:**
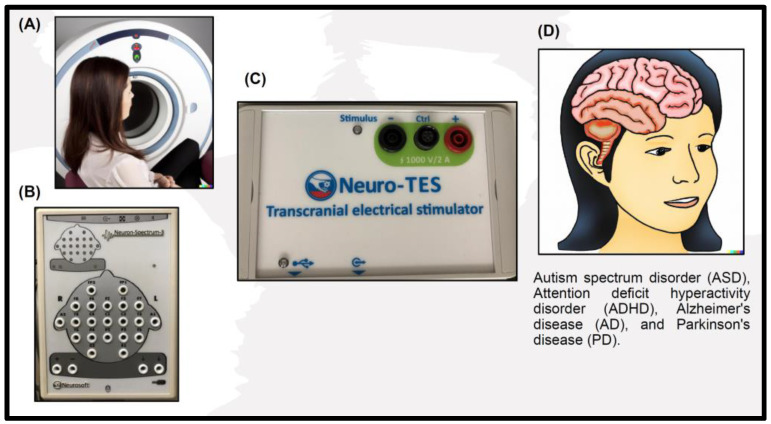
Neuroimaging techniques such as fMRI (**A**) and EEG (**B**) have revolutionized our understanding of brain function and have become essential tools in studying neurological disorders. Furthermore, the review highlights the role of electrical stimulation (**C**) in diseases (**D**) such as ASD, ADHD, AD, and PD. Neuroimaging techniques have significantly improved our understanding of brain function and its application to studying neurological disorders. Pictures (**A**) and (**D**) are from DALL·E 2.

Recent advances in neuroimaging techniques have significantly impacted neuroscience research and clinical practice. Here are some of the most notable advances: High-resolution imaging: Advances in MRI technology have enabled the imaging of the brain with unprecedented resolution, providing researchers and clinicians with a more detailed understanding of brain structure and function. Multimodal imaging: The integration of multiple imaging modalities, such as fMRI and EEG, has allowed for a more comprehensive understanding of brain activity and has helped overcome individual techniques’ limitations. Connectomics: Connectomics, which involves mapping the connections between different brain areas, has provided insights into the neural networks underlying various functions and helped identify biomarkers for neurological and psychiatric disorders. Personalized medicine: The use of neuroimaging biomarkers has enabled more personalized approaches to medicine, allowing clinicians to develop tailored treatments optimized for each patient. Recent advances in neuroimaging have significantly impacted neuroscience research and clinical practice and provided new insights into the workings of the brain and the mechanisms underlying neurological and psychiatric disorders. tDCS has been investigated as a potential treatment for various neurological and psychiatric conditions, including ASD, ADHD, AD, and PD (Figure 1). While tDCS has shown promise as a potential treatment for various neurological and psychiatric conditions, more research is needed to determine its effectiveness and fully understand its underlying mechanisms of action.

Based on the current review, some clinical implications and recommendations can be drawn for integrating neuroimaging techniques into real-world clinical practice and medicine. First, the study highlights advances in fMRI and EEG techniques, emphasizing their utility in understanding brain function and disease. These techniques provide valuable insights into the underlying neural mechanisms of various neurological and psychiatric disorders. Clinicians can leverage these technologies to improve diagnostic accuracy, monitor treatment response, and develop personalized treatment strategies. Second, the review highlights the importance of interdisciplinary collaboration and integrated care. Neuroimaging techniques provide objective measures of brain activity that can be combined with clinical assessment, behavioral observations, and other diagnostic tools. This integration allows for a more complete understanding of a patient’s condition, facilitates tailored treatments, and improves patient outcomes. Furthermore, the review suggests that future research should focus on refining neuroimaging techniques and developing standardized protocols for their clinical implementation. This includes improving spatial and temporal resolution, addressing signal-to-noise ratio and artifacts challenges, and developing robust data analysis methods. By improving the accuracy and reliability of neuroimaging measurements, clinicians can confidently incorporate these techniques into routine clinical practice. The review highlights the need for multidisciplinary collaboration between clinicians, researchers, and imaging specialists regarding comprehensive care recommendations. This collaborative approach can facilitate the exchange of knowledge and expertise, promote the interpretation of neuroimaging findings, and facilitate a thorough understanding of a patient’s condition. In addition, the review highlights the importance of ethical considerations such as informed consent, privacy protection, and responsible use of neuroimaging techniques in clinical practice. In conclusion, this review highlights the clinical relevance of neuroimaging methods in advancing our understanding of brain function and disease. It emphasizes the importance of interdisciplinary collaboration, calls for further research and technological advances, and recommends integrating neuroimaging findings with clinical assessment to enable individualized and comprehensive patient care.

Neuroimaging techniques in research and clinical applications raise several ethical considerations and concerns. Here are some critical areas of concern: Privacy and confidentiality: Neuroimaging data can reveal sensitive information about an individual’s brain structure, function, and possibly even their thoughts or intentions. Ensuring the privacy and confidentiality of neuroimaging data is critical to protecting the rights of participants and preventing potential harm. Strict data security measures and anonymization techniques should be implemented to minimize the risk of unauthorized access or data leakage. Informed Consent: Participants in neuroimaging research should provide informed consent to understand the purpose, risks, benefits, and potential implications of the study. The nature of the imaging procedure, any possible discomfort or adverse effects, and the intended use of the data collected must be explained. Participants should also understand their rights regarding data privacy and how their data will be stored, analyzed, and shared. Ethical use of data: Neuroimaging data should be used responsibly and ethically. Researchers and clinicians must ensure that the data collected is used for its intended purpose and protected from inappropriate uses such as stigma, discrimination, or invasion of privacy. Data sharing should follow established guidelines and regulations to prevent potential misuse or unintended consequences. Social influence: Advances in neuroimaging techniques have raised the possibility of colonial power, including neurobiological determinism or reductionism. Misinterpreting or misrepresenting neuroimaging findings can lead to simplistic or deterministic interpretations of human behavior or mental states. The ethical responsibility lies in accurately communicating the limitations and complexities of neuroimaging research to avoid drawing unnecessary conclusions or negative societal repercussions. Addressing these ethical issues requires adherence to ethical guidelines, regulatory frameworks, and ongoing ethical discussions within the scientific community. Researchers, clinicians, and policymakers must adopt a responsible and transparent approach that safeguards the rights and welfare of individuals involved in neuroimaging research and ensures the ethical use of neuroimaging techniques in clinical settings.

While neuroimaging techniques have significantly improved our understanding of brain function and disease, gaps, and limitations still need to be addressed. Here are some areas where future research and exploration can help fill these gaps: Complex brain networks: Neuroimaging provides insight into functional connectivity and structural networks in the brain. However, our understanding of these networks’ intricate interactions and dynamics remains limited, including their development, plasticity, and dysfunction in various diseases. Future research could focus on unraveling the complexity of brain networks, exploring the role of network connectivity patterns in cognitive processes and neurological diseases, and identifying biomarkers that could facilitate early detection and personalized treatment approaches. Multimodal integration: Neuroimaging techniques often provide complementary information about brain structure, function, and connectivity. Integrating multiple modalities, such as fMRI, EEG, DTI, and PET, can enhance our understanding of brain disorders by combining spatial, temporal, and molecular information. Future research could focus on developing robust methods for multimodal data integration and analysis to comprehensively understand brain function and how it changes in various diseases. Translation to Clinical Application: While neuroimaging research has improved our understanding of brain disorders, there is still a need to bridge the gap between research findings and clinical application. Future studies could focus on validating neuroimaging biomarkers, establishing their diagnostic and prognostic utility, and evaluating their effectiveness in guiding treatment decisions and monitoring treatment outcomes. This translational research helps ensure that neuroimaging techniques immediately impact clinical practice and patient care. By focusing on these areas for future research and exploration, we can further enhance our understanding of brain function and disease, paving the way for improved diagnostic tools, targeted interventions, and advances in neuroscience.

There are several emerging neuroimaging techniques and techniques that promise to advance our understanding of brain function and disease. Here are some examples: Magnetoencephalography (MEG): MEG measures the magnetic fields produced by the electrical activity of neurons in the brain. It provides high temporal resolution and can capture brain dynamics down to milliseconds. MEG is beneficial for studying neural oscillations and functional connectivity, gaining insight into brain networks and their disruption in disorders such as epilepsy, ASD, and ADHD. Functional near-infrared spectroscopy (fNIRS): fNIRS is a non-invasive imaging technique that measures changes in blood oxygenation in the brain. It uses near-infrared light to assess brain activity. fNIRS is portable and can be used in various settings, making it suitable for studying brain function in natural environments. It shows promise in applications such as cognitive neuroscience, neurorehabilitation, and brain-computer interfaces. Connectomics: Connectomics aims to map and understand the complex network of connections within the brain. It combines techniques such as diffusion MRI, resting-state fMRI, and graph theory analysis to study the structural and functional connectivity of the brain. Connectomics provides insights into the organization and integration of brain networks and has the potential to reveal connectivity changes associated with various neurological and psychiatric disorders. These emerging neuroimaging techniques and techniques have the potential to expand our understanding of brain function and conditions by providing improved spatial and temporal resolution, assessing neurochemical profiles, capturing dynamic brain activity, and revealing complex brain networks. These technologies’ continued advancement and application will likely facilitate significant advances in neuroscience research and clinical applications in the coming years.

Neuroimaging techniques have had a transformative impact on neuroscience, revolutionizing our understanding of brain function and disease. These techniques give researchers a window into the human brain, enabling non-invasive studies of its structure, function, connectivity, and metabolism. Significant contributions and advances in the application of neuroimaging techniques include Mapping brain function: Functional neuroimaging techniques, such as fMRI and EEG, allow researchers to map brain activity during various cognitive tasks, revealing language and emotion. These findings significantly advance our understanding of brain function and the complex networks that support complex cognitive processes. Uncovering Brain Disorders: Neuroimaging has played a crucial role in elucidating the neural mechanisms of neurological and psychiatric disorders. Neuroimaging research has identified structural and functional abnormalities associated with AD, PD, schizophrenia, depression, and autism spectrum disorders. This knowledge improves diagnostic accuracy, guides treatment strategies, and provides insights into the underlying pathology of these diseases. Studying brain connectivity: Neuroimaging techniques facilitate the exploration of brain connectivity and network organization. The combination of fMRI, DTI, and other modalities allows researchers to map complex neural networks and study how they are altered in neurological and psychiatric disorders. Understanding brain connectivity improves our knowledge of how different brain regions interact and cooperate, providing insights into information processing, cognitive function, and changes in disease-associated connectivity. Translational application: Neuroimaging techniques show promise in translational applications, bridging the gap between research and clinical practice. For example, imaging-based biomarkers have been explored for early diagnosis, prognosis, and prediction of treatment response in various diseases. In addition, neuroimaging-guided interventions, such as TMS and TES, have emerged as potential therapeutic tools for brain disorders. The application of neuroimaging techniques has dramatically advanced our understanding of brain function and provided important insights into the underlying mechanisms of neurological and psychiatric disorders. These technologies open new avenues of research, improve diagnostic accuracy, guide treatment strategies, and facilitate the development of novel therapeutic interventions. As technology advances, neuroimaging techniques promise to enhance our understanding of the brain further and help develop personalized medicine and interventions for brain disorders.

## Figures and Tables

**Table 1:** Some evidence suggests that tDCS may be beneficial in improving social communication deficits in individuals with ASD.

Study	Effects	Reference
Auvichayapat, P. et al. (2022)	The main objective of this paper is to investigate the lasting effects of tDCS on the treatment of patients with ASD. The study utilized a randomized controlled trial design, with the control group receiving sham stimulation and the experimental group receiving tDCS treatment. The study evaluated the efficacy of treatment at 6 and 12 months post-treatment. The findings suggest that compared to the control group, the experimental group who received tDCS demonstrated significant improvements in symptoms of autism, language, and social interaction 6 and 12 months after treatment.	[95]
D’Urso, G. et al. (2021)	Research focuses on cerebellar transcranial direct current stimulation’s efficacy, feasibility, and safety in children with autism spectrum disorders. The study also explored unexpected results, such as the impact of tic disorders and epilepsy in these children.	[90]
Kang, J. et al. (2023)	The main objective of this article is to explore the potential impact of tDCS on ASD by examining differences in EEG microstates between typically developing children and those with ASD. Moreover, the study aimed to compare EEG microstates and scores on the Autism Behavior Checklist before and after tDCS in children with ASD who were given either experimental or sham stimulation. The results indicate that tDCS could be a promising intervention for ASD.	[99]
Prillinger, K. et al. (2021)	The main objective of this paper is to outline the study protocol for a double-blind, randomized, and sham-controlled clinical trial that seeks to examine the impact of repeated tDCS sessions on adolescents who have ASD. The paper includes comprehensive information on the study design, criteria for participant selection, intervention procedures, outcome measures, and statistical analysis approach.	[93]
Qiu, J. et al. (2021)	This article centers on investigating the impact of tDCS on the left dorsolateral prefrontal cortex on cognitive and behavioral functioning among children with ASD. Specifically, the study aims to determine whether tDCS can enhance social communication, executive function, and behavior among children diagnosed with ASD.	[94]
Sun, C. et al. (2022)	This paper aims to evaluate the effects of tDCS, one of the first-line treatments, on neuronal activity in children with autism. Specifically, the study explored the impact of tDCS on the MMN (mismatch negativity) response characteristics of brain-dysregulated stimuli in children with autism, which is related to the perception and language development of children with autism. The study used quantitative electroencephalography (QEEG) technology to measure brain electrical activity and compared tDCS with sham stimulation (sham) to assess its effect on MMN responses.	[97]

**Table 2:** There is some evidence to suggest that tDCS may be beneficial in reducing symptoms of ADHD.

Study	Effects	Reference
Barham, H. et al. (2022)	This research aims to explore the possibility of using tDCS to enhance executive function, with a focus on planning and working memory, in adults diagnosed with ADHD. Through conducting five consecutive sessions of neuropsychological tests, the study aims to compare the effectiveness of active tDCS and sham stimulation. The study concludes that tDCS can be an effective method to modulate cognitive functions in adults diagnosed with ADHD.	[100]
Dubreuil-Vall, L. et al. (2021)	The study finds that anodal tDCS targeting the left dorsolateral prefrontal cortex modulates cognitive and physiological measures in the Eriksen flanker task in a state-dependent manner, suggesting that tDCS has a positive effect on cognition in ADHD. Furthermore, the study highlights the importance of event-related potentials as cross-sectional biomarkers for executive performance and their implications for developing personalized treatments for ADHD.	[101]
Guimaraes, R.S.Q. et al. (2021)	The study utilizes a randomized, triple-blind, sham-controlled crossover design to assess the therapeutic effects, as well as the safety and feasibility of tDCS treatment. Multiple neuropsychological tests, including the Wechsler Intelligence Scale and the Neuropsychological Assessment Battery, will be administered to assess patient performance before and after tDCS treatment. The study’s outcomes may facilitate the development of alternative, non-pharmaceutical treatments for enhancing cognitive and behavioral performance in patients diagnosed with ADHD.	[102]
Nejati, V. et al. (2021)	This research aimed to investigate the impact of tDCS on inhibitory control in children with ADHD and assess whether the severity of ADHD symptoms influenced the effectiveness of tDCS. The study involved two groups of children with ADHD, one with severe and the other with mild symptoms, who underwent anodal or sham tDCS over the right dorsolateral prefrontal cortex while performing inhibitory control tasks.	[104]

**Table 3:** Some evidence suggests that tDCS may be beneficial in improving cognitive function in individuals with AD.

Study	Effects	Reference
Andrade, S.M. et al. (2022)	The research is a double-blind, randomized clinical trial involving 36 AD patients. The results demonstrated that anodal tDCS + CS enhanced overall cognitive function and altered EEG brain activity in comparison to sham tDCS + CS, and changes in cognitive performance were related to modifications in EEG measures of brain activity.	[127]
Rasmussen, I.D. et al. (2021)	The objective of this research paper is to explore the impact of personalized HD-tDCS on memory performance and brain structure in individuals with AD. The study utilized computer modeling based on each patient’s MRI to determine the most effective electrode placement for optimal treatment outcomes. The findings indicated that different electrode placements yielded the best outcomes for each individual patient. Furthermore, the study revealed that after receiving HD-tDCS treatment, participants in the experimental group demonstrated significant enhancements in delayed memory and Mini-Mental State Examination (MMSE) scores. The results also showed a significant positive correlation between the strength of the electric field in the prefrontal cortex and memory improvement in the experimental group.	[129]
Saxena, V. et al. (2021)	The analysis included 11 studies of high quality, and the findings demonstrated that tDCS significantly improves cognition in AD compared to placebo treatment. Furthermore, anodal tDCS was observed to be more effective than cathodal and dual stimulation. The meta-analysis concludes that tDCS, particularly anodal tDCS, is a well-tolerated and effective intervention for treating AD.	[130]

**Table 4:** Some evidence suggests that tDCS may be beneficial in improving motor symptoms in individuals with PD.

Study	Effects	Reference
Aksu et al. (2022)	Specifically, the study aimed to investigate the effectiveness and neural mechanisms of tDCS on cognitive functions in PD. The study involved 26 individuals with PD who received ten sessions of either active or sham tDCS applied over the dorsolateral prefrontal cortex twice daily for five days The results of the study showed that active tDCS led to improvements in delayed recall and executive functions and increased N1 and NoGo N2 amplitudes, which are believed to reflect attention, discriminability, cognitive control, and conflict monitoring. Overall, the article suggests that tDCS may have therapeutic potential for PD by improving cognitive control, episodic memory, and underlying neural mechanisms.	[132]
Mishra and Thrasher (2022)	The study used a double-blind, cross-over, and sham-controlled design and included 20 participants with PD. The findings indicated that combining DLPFC stimulation with task performance resulted in improved cognitive performance, which persisted even after tDCS treatment had ended. However, there was no significant impact on mobility.	[134]
Wong et al. (2022)	The study randomly assigned 36 participants to four groups: primary motor cortex tDCS, dorsal lateral prefrontal cortex (DLPFC) tDCS, cerebellum tDCS, or sham tDCS. The results indicated that all tDCS groups showed significantly improved dual-task gait speed compared to the pre-test. Still, the DLPFC tDCS group exhibited the most critical advance in dual-task walking speed and an increase in the silent period compared to the M1 tDCS and sham tDCS groups.	[136]

## Data Availability

Not applicable.
